# Draft Genome Sequence of *Lactobacillus reuteri* CECT8605

**DOI:** 10.1128/genomeA.00297-17

**Published:** 2017-05-04

**Authors:** Ana I. Sañudo, Mónica M. Olivares, Oscar Bañuelos

**Affiliations:** Research Department, Biosearch S. A., Granada, Spain

## Abstract

*Lactobacillus reuteri* CECT8605 has shown potential probiotic properties in both *in vitro* and *in vivo* assays. Besides its beneficial characteristics, general aspects concerning genetic stability and safety for human consumption have been studied. Its genome sequence has been a useful tool to support preliminary conclusions based on empirical observations.

## GENOME ANNOUNCEMENT

*Lactobacillus reuteri* CECT8605 has exhibited several biological activities with promising applications in the field of human health (1, 2). However, such beneficial characteristics are not the only important factors in selecting a microorganism as a possible probiotic. The genetic stability of microorganisms, determined in many cases by the presence of active prophages or extrachromosomal genetic elements, as well as their resistance to antimicrobial agents, are important aspects that determine their technological feasibility and its safety. The genome of *L. reuteri* CECT8605 was sequenced in order to characterize this microorganism in these directions in more detail.

The draft genome sequence of *L. reuteri* CECT8605 was determined using a 250-bp paired-end library with Illumina MiSeq technology (Illumina, USA) at GenProbio SRL (Cadorago, Italy). A total of 1,633,356 reads were generated and assembled into 207 contigs using MIRA version 4.0.2 with a coverage of 150-fold. Quality of the final contig assemblies was improved using the Burrows–Wheeler aligner, SAMtools suite, VarScan version 2.2.3, and GATK software package version 2.8-1. Offline open reading frame (ORF) prediction was performed with Prodigal version 2.6 and its automatic annotation by means of BLAST comparison against the NCBI databases and HMMER against the PFAM database. To identify sequences corresponding to rRNA and tRNA genes, RNAmmer version 1.2 and tRNAscan-SE version 1.21 were employed, respectively. The final sequence is composed of 2,333,530 bp, which contains 2,418 putative ORFs and a G+C content of 38.9%.

Several attempts were made to isolate plasmidic DNA, to induce lytic phase, and to detect free viral particles in *L. reuteri* CECT8605, but they yielded negative results (data not shown). *In silico* analyses with genome sequences supported empirical results: similarity searches revealed the absence of plasmid replicon sequences (3) and the presence of seven regions containing viral-related sequences (4), but with genetic structures probably not functional as active prophages.

On the other hand, among clinically relevant antibiotics,* L. reuteri* CECT8605 is resistant only to ampicillin. This phenotype could be due to the presence of β-lactamase(s) and/or penicillin-binding proteins (*pbp*) with low affinity against ampicillin. Although three ORFs were annotated as putative β-lactamases in the sequencing study, a specific search for antibiotic resistance sequences (5) did not identify any ampicillin resistance gene. On the contrary, the sequence of 1 (*pbp*2x) out of 5 putative *pbp* genes located in the *L. reuteri* CECT8605 genome showed a point-mutation (encoding Thr instead of Ala in amino acid 526 of the B5D07_03460 ORF). This mutation is related to ampicillin resistance in other *L. reuteri* strains and is considered nontransmissible (6). The genomic region where *pbp*2x is found in the *L. reuteri* CECT8605 genome contains other genes involved in peptidoglycan synthesis and cell division, but does not contain genetic mobile elements. These preliminary results seem to indicate that a transfer event of ampicillin resistance to other microorganisms is unlikely to occur.

### Accession number(s).

The complete genome sequence of *L. reuteri* CECT8605 has been deposited in GenBank under accession number MWVS00000000 (BioProject no. PRJNA377846).
